# A Communication-Ecological Account of Groups

**DOI:** 10.3389/fpsyg.2021.797544

**Published:** 2022-02-10

**Authors:** Robin Kurilla

**Affiliations:** Institute of Communication Studies, Faculty of Humanities, University of Duisburg-Essen, Essen, Germany

**Keywords:** group, communication, collective identity, group identity, social theory, ethnography, environment, ecology

## Abstract

This article presents a novel conception of groups and social processes within and among groups from a communication-ecological perspective that integrates approaches as different as Garfinkel’s ethnomethodology, Heideggerian praxeology, and Luhmann’s systems theory into an innovative social-theoretical framework. A group is understood as a social entity capable of collective action that is an object to itself and insofar possesses an identity. The elementary operations of groups consist in social processes with communicative, pre-communicative, and non-communicative episodes. Groups operate in a number of environments that are conceived of as both correlates of their own processes and providing groups with the raw materials for the fabrication of their constituents. These environments include but are not limited to spatial, discursive, emotional, institutional, semiotic-medial, psychic-personal, technical, and groupal environments. The article paves the way to combine studies on intergroup and intragroup communication in one comprehensive theoretical framework situated on such an abstract level that it can be concretized in view of utterly different cultural contexts and the emic perspectives of actors therein. Accordingly, the framework provides researchers with the conceptual devices to balance the comparability of different lifeworlds with the faithfulness to actors’ inside views. The methodological implications laid out in this article prioritize qualitative, especially ethnographic methods as a starting point for research on group communication.

## Introduction

It is certainly not a coincidence that a publication (Verbeck, 2001) on increasing efficiency through team management praises “group research” as a trait of the 20th century. Not only academic studies (re-)discover groups as a subject. Everyday life in general experiences a group euphoria. Schools and universities celebrate learning groups, while post-Fordist companies implement and encourage team work. Despite the common designation, “group research” is everything but homogeneous. In humanities and social sciences, neither a common terminology nor a consensus regarding a general concept of groups have been established.

Even within the field of group communication, there is no consensus regarding the theoretical base or the phenomenal range. Individual studies operate in different paradigms. As a result, theoretical and empirical insights are not organized in a unifying conceptual frame, which makes communication among scholars difficult, if not impossible. At least, a number of common flaws of different approaches can be identified.

Firstly, there is a tendency to consider shared aims, norms, and/or values the defining characteristics of groups. This might be faithful to the self-descriptions of certain groups such as groups that operate in corporate contexts. The *a priori* assumption of all groups bearing these characteristics, however, may be a product of ethnocentrism. Non-reflective theorizing may lead to a similar bias in the sense that the researchers’ own relevance structures and political, economic, cultural, etc., situatedness are not explicated, further contributing to an objectivistic or universalistic understanding of groups.

Secondly, a Cartesian or cognitivist bias obscures pre-reflective processes that underlie the constitution and operating of groups. In no study of group communication, pre-reflective processing is consequently taken into account, which involuntarily leads to a misrepresentation of everyday life reality where reflective processes as represented by propositions and logical operations are everything but the norm. Inter-corporeal, embodied, tacit, emotional, and other layers of social reality are simply disregarded. Moreover, many studies bear a tendency to depict groups from a rational choice point of view, which forces group phenomena into a tight conceptual corset leading to a disregard of socio-cultural differences.

These two points often lead, thirdly, to a preference of experimental group research that does not pay attention to emic views. Fourthly, even approaches that do consider the constitution of boundaries and the groups’ relations to their contexts can be biased by the theoretical preferences researchers have, as a result of which the aporiae of structuration or rational choice theory may act as hidden determinants of research results.

Groups may, fifthly, be considered as functional units of society that operate in accordance with specific communication modes such as personal communication. Everyday perspectives are functionalized to explain the workings of society while the constitution and inner logic of groups are neglected. While communication is societally overdetermined here, it is conceptually underdetermined or completely undefined by many studies of group communication. This leads, sixthly, to a variety of epistemological and theoretical limitations of research on group communication.

This article presents a novel, communication-ecological model of groups intended to introduce a new theory of group communication that avoids the outlined pitfalls while conserving the benefits of the different approaches. Research on communication in small groups and research on intergroup communication will be united in one overarching model that helps to establish a comprehensive framework to organize the phenomenal scope through unified conceptual distinctions. Different paradigms of intergroup and intragroup communication will be presented and social processes will be determined that influence communication processes.

Building on approaches as different as Garfinkel’s ethnomethodology, Heideggerian praxeology, and Luhmann’s systems theory, a communication-ecological model is developed that is able to conceptually integrate a broad range of empirical phenomena while paying close attention to the emic views of groups and individuals. The model serves as an innovative comprehensive theoretical framework for group communication research. A group is understood as a social entity capable of collective action that is an object to itself and insofar possesses an identity. The elementary operations of groups consist in social processes with communicative, pre-communicative, and non-communicative episodes. Groups operate in a number of environments that are correlates of their own processes and provide groups with raw materials for the fabrication of their constituents. These environments include but are not limited to spatial, discursive, emotional, institutional, semiotic-medial, psychic-personal, technical, and group environments. This ecological model is faithful to inside views but also offers a base for comparisons of different lifeworlds. It acts as a theoretical framework situated on such an abstract level that it can be concretized across social and cultural differences. This way, the model can be employed in the “empirisch begründete Theoriebildung” (Kelle, 1997) inspired by Strauss and Corbin (1998) and Glaser and Strauss (2009) and thus supports the “quasi-inductive evolution of science” (Popper, 2002). It sensitizes researchers for cultural differences and prevents positivist self-misconceptions, which will be discussed in the section “Methodological Implications.”

To reach its aims, the article starts with a deconstruction of existing approaches to communication in small groups and intergroup communication (section “Approaches to Group Communication”). Especially their phenomenal scope and theoretical preconceptions are scrutinized. The approaches addressed are functional theory, symbolic convergence theory, structuration theory, the bona fide perspective, the social-psychological approach to intergroup communication, and systems-theoretical conceptualizations of groups.

The concept of groups will be introduced in the subsequent section “Groups.” Due to its centrality for the definition of groups, a multifaceted concept of identity will be developed thereafter (section “Identities”). The ensuing section “Social Processes and Group Communication” is dedicated to the conceptualization of social processes, particularly different types of group communication that are constitutive for groups. The ecological model employed to depict groups as well as social processes in and among groups will be introduced in the section “Environments of Group Communication,” whereas the methodological implications of the presented approach will be addressed in the section “Methodological Implications.” The article concludes with a discussion of its findings (section “Discussion”).

## Approaches to Group Communication

The approaches to be deconstructed in this section have been chosen for the simple reason that they explicitly focus on both groups and communication and are as such discussed in different branches of communication studies and neighboring disciplines. Such a selection necessarily excludes some works that are not irrelevant to group communication or can be considered interesting addresses for valuable comparisons with the theory developed here. The micro-sociological tradition (e.g., Goffman, 1966, 1967; Collins, 2004) provides interesting insights in social interactions but has not produced a coherent group concept and is not concerned with conceptual issues of communication. Hansen’s (2009) rich and highly nuanced work on collectives bears a lot of valuable insights for the study of relations among collectives, but it completely abandons the group concept and is also not explicitly concerned with communication. Network theories such as the actor-network theory (Latour, 2005) bear a similar problematic. The inspiring studies on “communities of practice” (Lave and Wenger, 1991; Wenger, 1999, etc.) also abandon the group concept and focus primarily on learning, not on communication. To reduce complexity and for didactical purposes, these approaches, among others, could not be included in this section. To compensate this momentary blind spot, however, tribute to some of these approaches will be paid and constructive distinctions from the presented model will be drawn wherever appropriate in the subsequent sections.

### Functional Theory

The functional theory of group communication is “a normative approach to describing and predicting group performance that focuses on the function of inputs and/or processes” (Wittenbaum et al., 2004). It is based on three premises. Firstly, groups are considered goal-oriented entities. Secondly, group performance can be evaluated. And, thirdly, group performance is influenced by internal and external parameters.

Hirokawa and Gouran (1989) relate group performance to decision-making and identify five tasks that a group has to accomplish to optimize its decisions: (1) an adequate comprehension of the problem to be solved, (2) identifying the minimal requirements of a solution, (3) identifying relevant and feasible alternatives, (4) examining the alternatives in view of the solution criteria, and (5) choosing the alternative that best fits the needs of a solution (Wittenbaum et al., 2004). An additional task consists in developing practices to overcome cognitive, affiliative, and ego-centered limitations (ibid.).

Empirical studies focus on problem-centered, decision-making groups in laboratory contexts without a history that exceeds these artificial contexts (Hirokawa and Gouran, 1989). The problems that emerge in the action process are considered describable according to fixed parameters that can be solved in a rational way (ibid).

The functional theory bears a number of limitations. It excludes all aspects of group realities that are not related to an assumed group task. Informal relations that transcend the group interaction cannot be taken into account. Groups appear as objective entities with clear boundaries. The variables that are isolated in laboratory simulations are not necessarily relevant in social reality. Categories are determined without assuring that they are faithful to the inside views of the group. Correspondingly, the subject area is reduced to goal-oriented groups that make decisions according to the principle of rational choice.

Communication is not even explicitly conceptualized but seems to be understood as a type of propositional information processing – not on a psychical but on a social level. In other words, communication appears as a socially extended nervous system. This view comes close to the information-theoretical depiction of communication and, thus, bears the same epistemological pitfalls. This reductionist conception ignores the emergent characteristics of communicative processes.

### Symbolic Convergence Theory

Bormann (1985, 1996) considers his theory of symbolic convergence a general theory of communication with a transhistorical and transcultural scope. In Bormann’s (1985) own words, the theory addresses “the appearance of a group consciousness, with its implied shared emotions, motives, and meanings, not in terms of individual daydreams and scripts but rather in terms of socially shared narrations or fantasies.”

The theory of symbolic convergence examines repetitive forms and patterns of communication that are considered the outcome of the constitution of a group consciousness. It describes the dynamics that contribute to the development, maintenance, decay, and disappearance of this consciousness. A focus lies on “fantasies” supposedly shared by communicators. To Bormann (1996), such fantasies start with dramatizing messages that stimulate an empathic involvement and can be identified when communicators show emotional consonance regarding particular events. “The result of sharing dramatizing messages is a group fantasy; the content of the dramatizing message that sparks the chain of reactions and feelings is called a fantasy theme” (Bormann, 1985).

Bormann calls fantasies with similar courses of action “fantasy types.” Among these types, Bormann (1996) locates archetypical fantasies that group members easily recognize. “Rhetorical vision” refers to the more or less coherent integration of a group’s fantasy types and themes. To Bormann (1996), the constitution of rhetorical visions increases the formal inclusion and integration of individuals into the group.

Bormann’s theory surely provides the conceptual tools to describe and explain the reduction of contingency along thematic lines. Symbolic convergence is established when the same narratives, puns, rhetorical figures, etc., can be repeatedly observed in groups. Bormann (1985), however, goes a step further and confounds two logical levels, taking the effortlessness with which those patterns are reproduced as an indicator for the development of shared meanings. The coherence of social processes leads Bormann to believe that the underlying psychic processes are coherent, which serves as the foundation for postulating a shared consciousness, identity, emotions, etc. The theory of symbolic convergence turns out to be a theory of psychic convergence.

The Hegelian or social-ontological connotations this conception evokes are, however, misleading. Identity, consciousness, and emotion remain phenomena that are socially constituted but bound to individuals. Following Bales (1970), Bormann (1996) situates the initiation of fantasy themes in the psychodynamic concerns of individuals. This social-psychological limitation leaves no space to acknowledge the emergent character of communicative processes. Bormann’s theory overlooks that collective action does not depend on a shared consciousness or shared emotions. Supraindividual processes are obscured by the empty formula of a group consciousness which, in the end, is situated in the “mind” of individuals. The problem of the constitution of an emergent order is not addressed. As a result, Bormann falls behind his aspiration of offering a general theory of communication. His theory falls prey to the same epistemological aporiae as Gadamer’s (2010) concept of a fusion of horizons. Beside the emergence of communicative processes, pre-reflective practices constitutive for groups are disregarded.

### Structuration Theory

Giddens’ (1984) structuration theory has been employed in research on small groups. The duality of structure that Giddens postulates to reconcile objectivistic and interpretative approaches is translated into the “distinction between *system*, the observable pattern of relations in a group, and *structure*, the rules and resources members use to generate and sustain the group system. Structuration theory construes the observable group system as a set of practices constituted by members’ structuring behavior” (Arrow et al., 2004, see also Poole et al., 1996).

According to Poole (2013), research on groups inspired by structuration theory is characterized by a mix of quantitative and qualitative methods. That does not mean, however, that triangulations of methods are frequent. Giddens’ structuration theory rather establishes an illusionary consensus that allows researchers to remain in their own paradigms after paying lip service to the importance of the paradigms on the respective other side of the duality of structure. A true integration is not achieved (see Kurilla, 2007).

The tension between change and tradition may be acknowledged, but this does neither help to explain how external structures can shape action within groups if not as practices. Nor can be explained how structures could have an existence that is independent of the practices that constitute the structures.

The conception of rules and resources not as fabrications co-constructed from raw materials in interactions but as individual assets sheds light on two problems of structuration theory. Firstly, rules seem to be the product of a Cartesian cogito, which results in a cognitivist bias, since rules seem to be bundles of propositions. Secondly, the emergence and inherent logic of social processes are disregarded, and social change seems to be reduced to cognitive interpretations and the ensuing actions of individuals.

The subject range of the structuration theory exceeds the scope of the symbolic convergence theory. Not only fantasy themes orientate the constitution, maintenance, and change of social order, but, more generally, a duality of structure. Both theories, however, bear cognitivist, and social-psychological limitations.

The most significant shortcoming of structuration theory is the lack of an explicit concept of communication. Not even Poole et al. (1996) supposedly foundation-theoretical treatise of group communication, let alone the connected empirical studies present a communication concept. Implicitly, however, communication is treated as a rule-based process of coding, transmitting, and decoding of messages, which suggests that an information-theoretical communication model is employed.

### The Bona Fide Perspective

The bona fide perspective on group communication was first described by Putnam and Stohl (1990), Frey (2003). It offers an alternative to the container model of group communication research that depicts groups as hermitically sealed entities. Groups are characterized through two traits. Firstly, they have stable, yet permeable boundaries, and, secondly, they are interdependent with the multiple contexts they operate in (ibid., Putnam and Stohl, 1996).

Four factors are held responsible for the permeability of group boundaries: (1) memberships of individuals in different groups and resulting role conflicts, (2) members of a group that act as representatives of other groups, (3) influx and outflux of members and the resulting changes of internal group dynamics, and (4) the development of a so-called group identity (Putnam and Stohl, 1996; Frey, 2003). Putnam and Stohl (1996) also name four factors to explain the interdependence of groups and their contexts: “intergroup communication, coordinated actions among groups, negotiation of jurisdiction and autonomy, and interpretative frames for making sense of intergroup relations” (ibid., Stohl and Putnam, 1994; Frey, 2003).

The bona fide perspective has inspired a variety of studies with ethnographic aspects. Conquergood (1994), e.g., examines the organization of and relations among gangs from an emic perspective. He discovers organization structures and boundary practices that are surely faithful to the inside views of the individuals he studied. Conquergood misses, however, that the principles he describes do not necessarily translate directly in social practices. Moreover, communication is only depicted as a means to certain aims without determining the traits of communication processes. This might result from the theoretical abstinence of many bona fide studies and surely benefits ethnographic research. Unfortunately, this abstinence is not guided by a grand theory as a general frame of interpretation but rather undermined by *ad hoc* hypotheses from different approaches such as the structuration theory. Other studies framed as belonging to the bona fide perspective, e.g., Houston (2003), do not even try to apply ethnographic means but rely on second-hand accounts and tacit theoretical preconceptions.

In addition, the bona fide perspective bears the following limitations. Although Putnam and Stohl (1996) speak of “intergroup communication,” they do not provide any definition of communication. How complexity is built in the course of social processes remains undetermined. Some expressions elicit the impression that the implicitly employed communication concept suffers from the same constraints as the information-theoretical model: “group members ignored external information” or “information processing across boundaries.”

This conception might also stem from an implicit orientation on rational choice theory. The context included in the considerations is often the context of formal organizations as the bottleneck of the societal influence on groups. A closer examination of contexts on a general level is missing. A further, cognitivist limitation lies in the disregard of supraindividual processes. It might be acknowledged that groups interact with external contexts, but the boundaries to those contexts are depicted as being objectified. Pre-reflective, practical boundaries do not conceptually enter the perspective. There is also a social-psychological limitation with regard to the concept of identity. Group identities are treated as individuals’ mental representations of their belonging to groups (Oetzel and Robbins, 2003). Group identities are thus reduced to a consensual area in the minds of individuals, not as identities of social entities. Boundaries and identities are only communicatively triggered but established mentally as cognitive phenomena.

### Intergroup Communication

Unlike the approaches discussed so far, the following research tradition is based on a rather broad group concept (like Simmel, 1908). The major influence on the paradigm of “intergroup communication,” however, is the theory of social identity (Tajfel, 1974, 1982b; Tajfel and Turner, 1979; Turner, 1982). Counterintuitively, the subject of this tradition is “not communication that occurs between groups. Rather it occurs when the transmission or reception of messages is influenced by the group memberships of the individuals involved” (Gudykunst and Lim, 1986; Harwood et al., 2005).

Accordingly, intergroup communication takes place whenever people are addressed not as persons, but as exemplars of social categories, which is equated with being a member of a group. Whenever a man addresses a woman as a woman, whenever a black person addresses a white person as a white person, whenever a patient addresses a physician as a physician, we are faced with intergroup communication according to this definition. In turn, when people are not addressed as members of a group, i.e., when no social category or, to use Tajfel’s term, social identity is ascribed to them, but they are addressed as persons, it is called “interpersonal communication.”

There is no doubt that the ascription of social categories can orientate communication. It is, however, problematic to talk about these cases as if it would be communication between groups or members of different groups. Firstly, social categories can be used to form groups – a group of female employes of a company, a group of fathers, of mothers, etc. All men, all women, all fathers, all mothers, etc., however, do not form an interconnected unit, let alone a social entity capable of collective action. Social categories rather designate “imagined communities” (Anderson, 2006) than groups. Secondly, social categories are not regarded as being co-constructed through interaction. Thirdly, the way Tajfel et al. conceptualize social identities seems to be too objectivistic. It is not even clear that in the inside views of all collectives, a seemingly basic concept as the one of mother is conceived of in the same way. For the Walbiri and the Tiwi, e.g., childbirth is not related to sexual intercourse but to dreams that sometimes act as a means of reincarnation (Meggitt, 1965; Herrmann, 1967; Hart and Philling, 1979). As a result, the role of mothers is different, and fathers are interchangeable with relative ease (Hart and Philling, 1979).

The mere ascription of different social categories to interactants is *not* considered intergroup communication in this article. Some aspects of this research direction, however, seem relevant to group communication research, but rather as interpersonal or intragroup communication. Families, e.g., are considered an “intergroup domain” by Soliz (2010), which translates here into intragroup communication influenced by the ascription of social categories. Likewise, most phenomena described by this research direction (discrimination, racial bias, etc.) often occur on the level of interpersonal communication understood as communication among individuals, not among empirically detectable groups. When persons are addressed as persons and not concerning the ascription of social categories, it will be termed “personal communication” that can occur in and among groups as well as among individuals.

### Systems Theory of Group Communication

Interestingly, personal communication becomes the defining criterium for groups in newer advances of Luhmann’s systems theory. In order to depict this conception, some general remarks on Luhmann’s theory are necessary.

Luhmann (1999) understands social systems as autopoietic systems. An autopoietic system produces and reproduces its elements only out of its own elements. In the case of social systems, the basic unit is communication. Only communication communicates, not persons. To Luhmann, the everyday life description of communication as actions performed by actors is a functional reduction of complexity that helps communication to proceed from one process to the next.

Contrary to communicative self-descriptions, the systems-theoretical viewpoint comprehends communication as an only recursively observable fusion of three selections: information, message, and understanding. Unlike in speech act theory (Austin, 1962; Searle, 1969), e.g., communication is not propelled by messaging intentions but by the ascription of messaging intentions. Alter sees that ego raises his hand, ascribes a messaging intention, and reacts by saying “hello.” It does not matter if ego indeed wanted to greet alter or merely scratch his head. Once verbal speech is used, it is difficult not to ascribe a messaging intention. As a result, ego becomes involved in communication although he might only say, “do we know each other?”

The communicative response to an action or behavior is called “understanding.” In our example, the word “hello” is the place of understanding that introduces the difference between message and information. The word “hello,” however, is not communicative as long as nobody replies to it communicatively, that is, responds to it in an observable way, making it recognizable as the difference between message (how) and information (what). In our example, this response was the phrase “do we know each other?”

Understanding does not mean the psychical understanding of meaning. Even the utterance “I do not understand what you are saying” is understanding in a communicative sense, as it indicates that the listener has noticed that an information was messaged. To Luhmann, understanding is a purely social operation that renders a preceding behavior part of a communicative process.

In communication, at least two psychic systems contribute to the constitution of a suprasystem. Communication is, however, not the only form of contact that individuals entertain. They also observe each other without ascribing a messaging intention. In most cases, I would not infer that someone wants to tell me something when I observe stains of red wine on a person’s shirt. This observation can, nevertheless, irritate communication.

Communication is reliant on perception and thus on psychic systems. Both system types, however, cannot directly interfere with each other’s mode of autopoiesis. Social systems consist only of communication and psychic systems only of thoughts (Luhmann, 1985). Both system types have co-evolved and use sense, preferably coded by language, which enables them to create environments to orientate their processes on.

Luhmann (1999) differentiates between three different types of social systems, each consisting of communication: interaction, organization, and society. The latter comprises all communications on a global scale. To Luhmann (1998), society has evolved through four stages with an increasing level of complexity. Nowadays’ functionally differentiated society consists of subsystems that are entrusted with one task that only they perform for society. Those functional subsystems comprise, among others, economy, politics, science, arts, education, and families.

Individuals are only partially included in the subsystems, particularly on the level or organizations. To Luhmann (1983), this ubiquitous partial inclusion leads to a novel need of a world of proximity. This need is satisfied with a new medium of communication: romantic love. Love is not considered an emotion but a symbolically generalized communication medium that serves to increase the probability of the acceptance of a communication offer like money in economics, power in politics, and truth in science. Love increases the chances that intimate communication is established. Love also serves as a code that prescribes that loved ones have to take each other into account in every decision they make – not regarding particular role expectations but as a whole person. In this sense, love creates the base for personal communication, communication that operates between persons, not their roles, in couples and families.

Families are not conceptualized as groups but as a subsystem of society. Luhmann (2000) himself is reluctant to use the term “group.” According to Wimmer (2012), Luhmann considers expressions like “group” or “team” as the epitome of a discourse that aims at semantically weakening the impact of hierarchies by rhetorically cherishing values like participation, equality, and self-fulfillment. Accordingly, the group concept becomes the equivalent of Tönnies’ (2005) notion of community as opposed to society. Luhmann’s successors largely ignored the concept of groups or, like Kieserling (1999), dissolved it by considering group phenomena either as mere chains of interactions or, once a certain level of complexity such as expressed by formal memberships has been reached, as organizations (see Kühl, 2021a).

After Luhmanns’s unpublished notes were made available, however, Kühl discovered another approach to groups in Luhmann’s early thinking. Kühl (2021b) considers groups as social systems that, like couples and families, are based on personal communication. It is expected that group members interact as persons and not according to role expectations which govern interactions in organizations where personal interactions are reduced to a minimum. Unlike in families, however, memberships in groups are highly contingent. Unlike organizations, groups are not thought of as having formal memberships but rather dynamic boundaries. Once memberships get formalized, a group ceases to be a group and becomes an organization. The paradigm par excellence for groups are friendships.

Luhmann’s systems theory and its understanding of groups bear a number of limitations. Everyday life accounts cannot be considered faithful descriptions of social reality but are treated as functional constructs that reduce complexity to maintain autopoietic processes. The horizon of Husserl’s phenomenology is reduced to a mere surplus of options for future operations. Sense becomes senseless, empty space. The trivialization of emic perspectives goes along with a disregard for cultural differences. Love can only be a global medium providing the universal foundations for romantic couples and families. Other forms of love are either considered residues of past societies or do not enter the considerations at all. Similarly, groups are based on personal relationships, no matter whether for individual group members the difference of formal organization and personal communication makes a difference. Luhmann’s theory also leaves no space to describe pre-reflective processes in another way than by the blind transition from one side of a scheme to another.

## Groups From a Communication-Ecological Perspective

In order to tackle the shortcomings while conserving the insights of the approaches discussed above and to unite the phenomena they aim at describing and explaining in one overarching theoretical frame, a communication-ecological model of groups is presented next. The concept of groups will be introduced first (section “Groups”). This first section raises questions regarding some components of the group definition that will be answered in the subsequent sections: The section “Identities” presents a conception of individual, collective, and group identities. In the section “Social Processes and Group Communication,” the social operations constitutive for groups will by described. The section “Environments of Group Communication” discusses the relations these processes entertain with their environments, which is the main focus of the communication-ecological model.

### Groups

The term “group” looks back on a long career in different disciplines like social anthropology (see Fuhse, 2006), sociology (Cooley, 1929; Bales, 1951; Freeman, 1992; Homans, 2010, etc.), group dynamics (Lewin, 1947; Forsyth, 2014, etc.), economics (most notably Olson, 1965), social psychology (Tajfel, 1974, 1982a, b; Turner, 1982, 1988; Hogg and Abrams, 1998; Sherif, 2015, etc.), communication studies (Hirokawa and Gouran, 1989; Putnam and Stohl, 1996; Harwood et al., 2005; Giles and Giles, 2012; Poole, 2013, etc.), sociometrics (Moreno, 1934, 1937), and cooperative game theory (e.g., Branzei et al., 2005). It would be a hopeless endeavor to find a common conceptual denominator that all research directions could agree on. There is, however, a less complex way to assure the adaptability of a communication-theoretical group concept. This way starts, following Kamlah and Lorenzen (1996), with the everyday life use of the term “group.”

As has been shown with Luhmann (2000), however, this starting point provokes skepticism. The group concept is often used to contrast society with community in the sense of Tönnies (2005), groups being identified with the latter. While Rousseau’s (2001) community of the *amour de soi-même* and Engels’s (1975) “ursprüngliche kommunistische Gesamthaushaltung” indeed evoke moralizing connotations, similar concepts such as Hegel’s (2002) “natürliche Sittlichkeit” and Simmel’s (1908) “kleine Kreise” do not carry moral implications. They are simply considered preliminary stages of societal development. Similarly, this article portrays the difference of society and community as a continuum where empirical groups can be located as more societal or more communal according to their degree of formalization. It thereby also avoids the other extreme of ethnocentrically reducing groups to societal entities that by definition have aims, norms, and/or values.

The common denominator of the everyday life use of the term “group” lies in the notion of “a number of persons that constitute an entity.” To emphasize the partial inclusion of individuals in modern societies’ social entities, the term “persons” will be replaced by the term “members.” A numerical maximum of group members is not determined while the numerical minimum is two. Simmel (1908), in turn, considers three members as the minimum to speak of a group, arguing that a collective of two persons would cease to exist if one person left and does thus not have a supraindividual character. This conception faces two major problems. Firstly, Simmel cannot answer what is left of a group of three if one person actually leaves the group. By definition, the group would already cease to exist, that is, lose its supraindividual character. Secondly, Simmel himself shows with the example of marriage that groups constituted by two members can bear a third element that establishes a more permanent character. By legal regulations, the institution of marriage formally includes the group into its societal vicinity and thus objectifies it.

The main reason to consider social entities with only two members as groups, however, is the fact that they operate according to their inherent logic. In our working definition, this is expressed by the specification “that constitute an entity.” Accordingly, “entity” does not mean that group members exist in atomistic isolation but that emergent social processes take place among them. Not the members, social processes form the basic units of groups. In the section “Social Processes and Group Communication,” these processes are discussed in detail. Unlike in Luhmann’s systems theory, however, these operations are not conceived of exclusively as communications but as social processes with communicative, pre-communicative, and non-communicative episodes.

Groups do not operate *in vacuo* but in a variety of environments that will be specified below (section “Environments of Group Communication”). Groups distil raw materials from these environments for the fabrication of their process components. Not only therefore, groups remain open entities despite their inherent logic. They are also open for interactions with other groups and external individuals and can orientate their operations on others. Otherwise, intergroup communication would be impossible and no group process could exceed the boundaries of groups. Groups may remain closed, however, on the level of their objectified identities.

Groups are principally able to perform collective actions, as they are constituted by supraindividual processes and can be treated as addresses of responsibility ascriptions, i.e., can be held accountable for their actions. Not every group performs collective actions. The defining criterium is not the empirical realization but the capability. Collective action does not require an explicitly drafted plan of action, even though in some groups such plans may play an important part. Communicative, pre-communicative, and non-communicative processes can also occur as purely pre-reflective practice.

Time is an important determinant of groups. In the following, the term “process history” is used to refer to the historical conditioning of group practices. A process history may transcend the borders of the group. No minimum amount of time to constitute a group is determined, since the unity of groups can only be recursively identified as such and expectations can be formed instantly. Groups can be transitory as well as durable. Temporality and contingency reduction will be discussed in detail below (section “Environments of Group Communication”).

Groups can be classified regarding potential contacts and actually realized contacts among members. The former will be called ‘‘degree of interconnectedness’’ and the latter ‘‘density of contacts.’’ The maximum degree of interconnectedness is given when all members can principally establish contact with each other. The minimum consists of members forming horizontally or vertically connected chains of contact^1^. When the density of contacts is high, members of a group interact frequently with each other; when it is low, interactions are less frequent.

The fundamental characteristic of a group is, however, that the unity of the group is present in the unit, that is, a re-entry, to use a Luhmann (1999) expression, has taken place. In other words, *the identity of a group is the defining criterium*^2^. Consequently, phenomena such as the constitution of order, processes of inclusion and exclusion, differentiation, change, e.g., are described and explained in the light of group identities. Identities are not to be confused with a ‘‘we feeling’’ or a ‘‘group mind.’’ The focus lies on communicative and pre-communicative processes that constitute the unit. Put in supposedly^3^ Marxian terms, a class in itself could not form a group, only a class for itself could. Recursively, however, similarities in everyday practices, everyday interactions, and life conditions could be considered “pre-adaptive advances” (Luhmann, 1998, 2005) of group constitution.

The following paraphrase of the working definition “a number of persons that constitute an entity” seems suitable: A group is a social unit capable of collective action that constitutes a unit for itself and insofar has an identity (see section “Identities”). As emergent phenomena the elementary operations of groups consist of social processes with communicative, pre-communicative, and non-communicative episodes (see section “Social Processes and Group Communication”). Groups can be transitory or durable. The numerical minimum group members is two, a maximum has not been specified. The degrees of interconnectedness and contact density vary among groups. Groups can be formalized to different degrees between the ideal-typical ends of the continuum of community and society. Groups operate in a variety of environments out of which they generate their process components (see section “Environments of Group Communication”).

### Identities

Due to their centrality in the definition of groups, identities have to be addressed next. George Herbert Mead who locates the constitution of identities in social processes delivers the blueprint for the concept^4^. Mead’s distinction of I and Me as two aspects of identity is generally interpreted through the lens of symbolic interactionism and the book “Mind, Self, Society” that was published by his former students. From this viewpoint, the I is identified with the biological, spontaneous, or uncontrollable articulation of the self. Goffman (1956) identifies the I with the person and the Me with her or his roles. There is, however, another reading of Mead’s distinction between I and Me that is derived from his own publications and rather epistemological than role-theoretical.

From Mead’s (1910) pragmatist-behavioristic perspective, the meaning of objects is determined by the reactions of the living beings that use those objects. Exploiting the double meaning of attitude as a body posture and a mental stance, Mead argues that the meaning of an object is acquired by taking on the attitude that others show toward the object. Generalization of meaning is achieved when an individual not only takes on the attitude of other individuals toward the object but the attitude that all others would show which Mead (1922) refers to as the “generalized attitude.” In this manner, not only the meaning of objects is obtained but also the meaning of the self as an object. The individual becomes aware of how others see it. The abstraction from individual differences is fostered by contexts of collective action, particularly competitive games such as football or baseball.

The Me is the self as an object, either from the perspective of single individuals or on a generalized level. The I, in turn, disappears in the blind spot of observation such as in Husserl’s (1976) phenomenology *noesis* or the process of experience can never be, simultaneously, the *noema* or the object of experience^5^. *Via* reflection, the I can only be grasped retrospectively as an object. In Mead’s (1913) own words, the translucence of the I is expressed as follows: ‘‘The, I’ of introspection is the self which enters into social relations with other selves. It is not the, I’ that is implied in the fact that one presents himself as a ‘me’. And the ‘me’ of introspection is the same ‘me’ that is the object of the social conduct of others. One presents himself as acting toward others -- in this presentation he is presented in indirect discourse as the subject of the action and is still an object -- and the subject of this presentation can never appear immediately in conscious experience^6^.”

Unlike Mead who only schematically differentiates between play and game, Vygotsky (1979) pays more attention to different empirical types of social action. This focus helps to develop a more practical-relational notion of the I. In this regard, Vygotsky paves the way for a conception of the pre-reflective side of self as rooted in social practice. Mead does not draw those conclusions, but his conception of the pre-reflective I could nevertheless be understood as governed by social practices. To conclude: On the level of individual identities, that is, identities of individuals, we distinguish between practical identities (I) and objectified identities (Me).

Now we address group identities that many authors reduce to aspects of individual identities. Building on his comparative studies of non-human primates (Tomasello, 2000; Tomasello et al., 2005), Tomasello (2009) presents a concept of group identities as products of cooperative action. Tomasello’s conception, however, bears two problems. Firstly, he follows social ontology (Tuomela and Miller, 1988; Gilbert, 1990, 1992, 2009; Searle, 1990; Tuomela, 1991), attributing we-intentions or shared intentions not as social ascriptions but as ontological facts to social entities. Secondly, despite the postulate of a group mind, the term “group identity” does not refer to the identity of a group but to the part of the individuals’ identities that is shaped by their group memberships, to social identities in the sense of Tajfel et al. (Tajfel, 1974, 1982a; Tajfel and Turner, 1979, etc.).

Differing from such conceptions, this article comprehends group identities as identities of groups that are not anchored in individual cognitions but carried by social processes (see section “Social Processes and Group Communication”). Group identities are “positive” facts. The continuity of group identities is not established by cognitive derivates but derived from the history of social processes, to which we will come back in more detail in the section “Environments of Group Communication.” Like the conception of individual identities, the conception of group identities follows Mead’s model. The Me of group identities is formed when the group sees itself from the perspective of outsiders or other groups. It is assumed that if there is no outsider or other group, there is no need to form a concept of oneself as a group.

History delivers a plethora of examples of how the identity of social units is introduced from the outside, e.g., in contexts of trade or war. The case of the Basque is very revealing. Coin finds from the first and second century BC lead Tovar (1987) to the assumption that the Spanish term “vasco” is not inspired by an autochthonous Basque word but has Celtiberian roots whose meaning is “highlanders” or “mountain people.” Similarly, the Basque term “euskaldun” seems to be rooted among the Auscer. Humboldt (2010) affirms that the Basque have “lost” the terms to designate their unity. Until the father of the Basque nationalism Sabino Arana delivers the needed neologisms at the end of the 19th century (Pagola Hernández, 2005), the Basque remain without their own words to refer to themselves as an entity.

Arana forges these symbolic materials to fabricate group identities not *ab ovo* but builds on already existing institutions such as the prefix ‘‘eusk.’’ Such materials will be termed ‘‘collective identities.’’ Collective identities turn into ‘‘group identities’’ when they are actually used by groups to fabricate their identities. These materials may, however, also be used by individuals to create their ‘‘individual identities.’’ The term ‘‘euskaldun’’ can be used by interacting individuals to designate the unity of their group as well as a person who individually identifies as Basque^7^.

**FIGURE 1:**
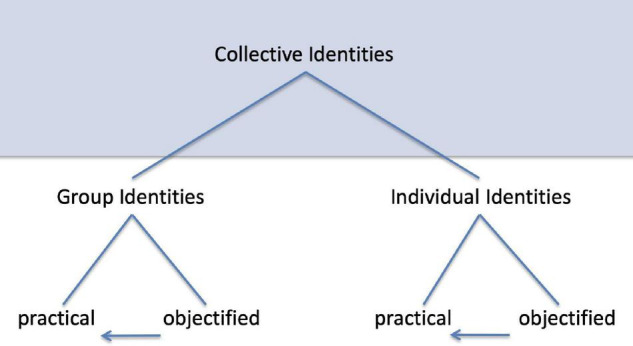
Relations among collective identities, group identities, and individual identities.

Collective identities are based on differences. These differences include cultural, political, national, age differences, etc. The genus “collective identity” bears the species of “cultural identity,” “national identity,” “corporate identity,” “gender identity,” etc. As raw materials, collective identities can be converted into the Me of a group, its objectified identity. Figure 1 shows the relations among collective identities, group identities, and individual identities^8^. Since collective identities belong to another logical type, they are depicted in front of a colored background. The arrows indicate that practical identities are only accessible as such after the fabrication of objectified group or individual identities. An example shall indicate the range and arbitrariness of objectified group identities.

Unlike Marx who divides the world into bourgeoisie and proletariat, the toilet paper brand Charmin makes a distinction between folders and scrunchers according to the way in which people conduct the cleaning process (Schramm, 2005). It is assumed on the basis of market research that people in the United States tend to be srcunchers whereas in Europe most people fold their toilet paper, which is relevant for product design. It is unlikely that people from the United States and Europe include toilet paper use in their self-descriptions or even in the depiction of cultural differences. This rather random distinction, however, is actually used to fabricate group identities as a look at internet forums reveals where people identify either as folders or as scrunchers and attribute character traits to both sides of the distinction^9^.

Even this example of individually performed practices shows that any practice can be objectified to serve as collective and group identities. Yet, particularly shared practices like laughing are prone to be employed in the fabrication of objectified identities. Even antagonistic practices like quarreling or fighting can provide the involved individuals with a foundation to identify as a unit. Simmel (1908) observes that a common enemy can unite individuals and groups even though their relations have been conflictive before. When a fight is interrupted by others, e.g., the people involved may well form a group of “fighters” against the external interruption. Since this is not very common, it is evident that practices can only be retrospectively, when objectified identities have been established, considered as practical identities. Otherwise, every shared practice would necessarily lead to the constitution of a group.

“Practical identities” are comparable to the I in Mead’s model. In relation to the fabrication of objectified identities, practical identities are pre-reflective. This does not mean, however, that they do not have any sense or meaning. Following Vygotsky and Tomasello, it is assumed that pre-reflective practices obtain their meaning from the action contexts there are situated in. Both aspects, pre-reflectivity and situatedness in action contexts, come together in Heidegger’s (1967) notion of *readiness-to-hand* on which the concept of practical identities is based. To Heidegger, being is not characterized by thinking but founded on a pre-reflective practice that is not objectified but *ready-to-hand*. The Cartesian and Kantian distinction of an observing subject and observed objects comes secondary in Heidegger’s thought. The reflective mind divides the world into subjects and objects primarily when practical problems arise. In Heideggerian terms, this Cartesian sphere of objectifications is the “presence-at-hand.” Objectified identities are situated here.

### Social Processes and Group Communication

This section addresses the social processes that are constitutive for groups. We will focus on the communicative and pre-communicative episodes of these processes and develop paradigms of intergroup and intragroup communication.

**FIGURE 2:**
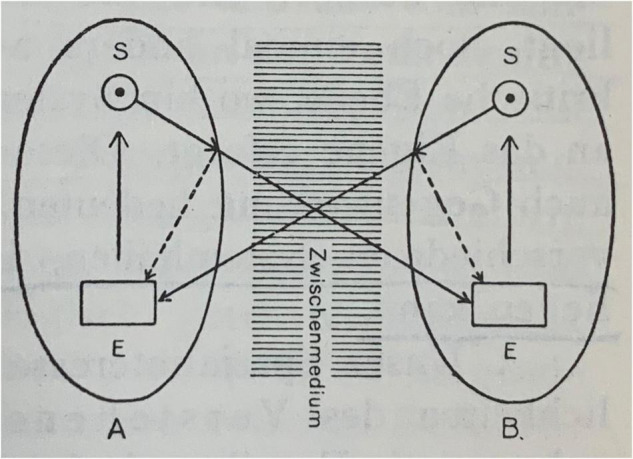
System of mutual guidance (Steuerung) between contact partners **(A,B)**, Bühler (1978).

Notwithstanding its manifold shortcomings, the mathematical model of communication by Shannon and Weaver (1964) has been highly influential in everyday life as well as academia and still persists in some currents of communication research^10^. A nowadays less popular alternative model with roots in Ancient Greece (Ungeheuer, 1987) that depicts communication as mutual guidance entered 20th century discourses through behaviorists concerned with language and communication such as Skinner (2014) and Bloomfield (1973). This model does not portray communication as a one-way street but as an interactive process based on feedback loops. Signals may be sent and received, but they are not coded and decoded according to a fixed set of probabilities but processed by the participants according to their own inner logic and might result in behavior, emotion, cognition, etc. Bühler (1978) presents such a cybernetic model of mutual guidance in 1927 already (see Figure 2)^11^.

The advantage of this model is that it emphasizes that communication follows its own logic that cannot be apprehended by considering either the speaker or the listener alone. Through Bateson, cybernetic models influenced the celebrated monograph “Pragmatics of Human Communication” by Watzlawick et al. (2011). Unlike Bateson or Bühler, however, these authors equate communication with behavior in interpersonal contexts, which is best expressed in their axiom ‘‘you cannot not communicate.’’ Accordingly, their model does not make a difference between, e.g., the observation of red wine stains on a shirt and a willingly communicated message such as ‘‘I have had a glass of red wine^12^.” In everyday life this difference makes a difference. Watzlawick et al. (2011), however, are only consequent when they do not refer to the speaker’s messaging intentions to distinguish between behavior and communicative acts. This step would have obscured the fact that communication processes produce their own supraindividual order that cannot be described and explained with reference to speaker intentions alone^13^.

There is, however, another method to include everyday life complexity into communication theory without undermining the inner logic of communication processes. This method turns to the listener (Schmitz, 1994, 1998) and, following Luhmann (1999), does not consider real messaging intentions of speakers as the propulsion of communication but messaging intentions attributed to speakers by listeners. This move helps to explain, why a person can be subjected to communication processes without having any intention to do so. Someone might scratch his head while another person thinks that it was a gesture of greeting and reply by saying “hi.” Quarrels that all participants want to solve but remain trapped in are another example of the emergent logic of communication processes that cannot be controlled by individual intentions.

These considerations result in the following communication concept: Communication is an emergent process of mutual guidance employing semiotic-medial devices that follows its own inherent logic between at least two personal or social entities and is propelled by the reciprocal ascription of messaging intentions. Communication serves the orientation and/or coordination in communicative and non-communicative action contexts. Individual communication episodes aim at listeners’ understanding. As correlates of its processing, communication produces a number of environments that, together with its process history, reduce the contingency of future processes (see section “Environments of Group Communication”). Simultaneously, these environments deliver the raw materials out of which communication synthesizes its process components and thus lay the foundations for its operations (see section “Environments of Group Communication”). Due to its emergent mode of operation, communication is conceived of as a unit that cannot be sufficiently described and explained by technological, cultural, semiotic, anthropological, etc., parameters alone.

We come now to the *pre-communicative episodes of social processes*. Communication is not the only form of interpersonal contact (Luhmann, 1985, 1995). There is also observation which will be defined simply as the perception or thematization of the conduct or actions of others without attributing a messaging intention. As Goffman (1966) shows, mutual observation and mutual reciprocal observation may create the basis of interactions under the condition of co-presence. There are many additional ways in which observation can influence communication. Observing the traces that tears have left on someone’s face, I might not tell the sad story I was originally going to. Whenever observation influences communication, it is a pre-communicative process. Other pre-communicative processes are, e.g., processes of study or practice to prepare communication offers.

**FIGURE 3:**
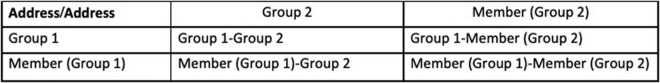
Paradigms of intergroup communication.

We have now all the conceptual means to define group communication. Like communication in general, group communication is propelled by the mutual ascription of messaging intentions. Logically, there are four paradigms of intergroup communication between two groups (see Figure 3). Firstly, groups might communicate as entities. This does not mean that groups actually have intentions such as messaging intentions like social ontology (Tuomela and Miller, 1988; Gilbert, 1990, 1992, 2009; Searle, 1990; Tuomela, 1991) would suggest^14^. The problems connected to this approach are avoided by focusing on the ascription of messaging intentions. Groups can act as addresses of communication and responsibility ascriptions, which in some cases even leads to legal consequences. Communication offers might be created by representatives of the group or by deliberating group members. The ascription of messaging intentions, however, can concern the group as a whole.

Secondly, a group as an entity might communicate with individual members of another group that in a given situation do not represent the group as a whole. The condition to designate this as intergroup communication is that individuals are actually addressed as members of the group. In turn, thirdly, members of the group might communicate with the other group as a whole. This is the exact opposite of the previous case. Fourthly, members of one group might communicate with members of another group. It is decisive that individuals involved in such communication are addressed as members of the involved groups. Messaging intentions are ascribed to individuals as members of the groups in this case.

The latter case may seem to belong to the phenomena of intergroup communication research as depicted above. This is, however, not the case, as it does not suffice to speak of ‘‘intergroup communication,’’ e.g., when a man addresses a woman as a woman or a black person addresses a white person as a white person^15^. Intergroup communication as depicted here only takes places when those social categories are actually used to fabricate groups that are constituted by social processes. From our perspective, most cases that are commonly referred to as intergroup communication are considered interpersonal communication or intragroup communication. It is likely, e.g., that conversations among a mother and a father of the same family are classified as intragroup communication rather than intergroup communication by the interactants themselves.

**FIGURE 4:**
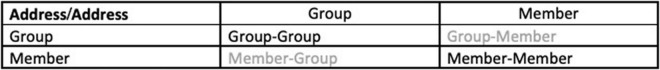
Paradigms of intragroup communication.

There are only three paradigms of intragroup communication (see Figure 4). The group can, firstly, communicate with individual members. This case resembles the intergroup communication between a group as a whole and members of another group with the decisive difference that the members of the own group and, respectively, the own group as a whole is addressed. This does, however, not create two paradigms of group communication, as communication is understood here as an at least two-sided process. For that reason, the two options of this case are colored in gray in Figure 4. Secondly, the members of a group might entertain intragroup communication. It is vital for this case that the members address each other as members of the group and not, e.g., as members of the board of two different organizations when the common base for intragroup communication would be the shared membership in a tennis club. And, thirdly, the group as a whole can communicate with itself as a whole. This might appear like a boundary case such as soliloquy. The difference to soliloquy, however, consists in the fact that actual communication processes can be observed with the factor time (see section ‘‘Environments of Group Communication’’) being an important component. A political party, e.g., might announce its aims and subsequently announce a change of its aims^16^.

### Environments of Group Communication

Communicative and pre-communicative processes distil their components out of their environments that, at the same time, are correlates of their operations. This conception is so central to the approach presented here that it is termed a “communication-ecological account.”

To Uexküll (1909), animals are not simply more or less successfully adapted to their environments. They are rather perfectly adapted because they themselves create their environments through their genetic blueprint. Similar to Uexküll, environments are considered correlative phenomena here. They are correlates, however, not of the interaction of a genetic blueprint with the surroundings of organisms but of communicative and pre-communicative social processes. Unlike in cybernetics with its conception of closed systems but similar to Bertalanffy’s (1950) theory of living systems, the relation of processes and their environments is depicted as an open one. Unlike in Bertalanffy’s account, this openness does not concern matter and energy in order to facilitate metabolism but raw materials situated in environments that are employed in the constitution of the components of social processes.

Like in Luhmann’s (1999) conception, environments are considered products of processes based on sense. Yet differing from Luhmann, sense is not only conceived of as objectified sense that is apprehended with two-sided schemes. To Luhmann, pre-reflective processes can only enter the consideration as blind changes from one side of a scheme to the other. In turn, the conception developed here depicts the relation to environments as meaningful in a double way. Environments can be accessed through objectifications, which is indeed comparable to Luhmann’s conception. In addition, environments are also accessed practically or pre-reflectively in the sense of Heidegger’s notion of the *readiness-to-hand* (see section “Identities”).

As correlates of past and current processes, environments are no material or ontic entities but epistemic tools of an observer. The model of environments as sources of raw materials for the production of process components helps to comprehend the constitution of order over time. The contingency of social processes is reduced on two levels. On the *practical level*, the process history that may result in habits or bodily dispositions (see Plessner, 1975; Bourdieu, 1995, 1996; Merleau-Ponty, 1995, etc.) renders more likely that practices that were performed in the past will be repeated in future processes. On the *level of objectifications*, logical or narrative coherence works against arbitrary changes of social processes. The latter, however, bears more room for ‘‘revolutions’’ than practical dispositions even though it remains unclear how the practice adapts to abrupt changes on the level of objectifications. The past is present in a twofold way in current processes, practically and thematically, as *dispositions generated by a process history* and as *its narrative objectification characterized by propositional coherence*^17^.

There is, however, a third factor that reduces the contingency of social processes. Social processes can and in many cases must transcend the boundaries of a group, unless a completely isolated tribe, e.g., is under consideration. As a result, the processes and their components have to be compatible with external processes. This external conditioning of social processes is the reason why in most instances group environments can be considered societal environments. Although the environments of social processes in groups are products of their own processes, they cannot be fabricated *ab ovo*. The socialization of members facilitates the adaptability of social processes in groups to external processes. For this reason, social realities of groups must be to a certain degree synchronized with their societal surroundings. As a result, the model presented here claims to be compatible with Garfinkel (1967, 1988, 1996, 2002) ethnomethodology that does not consider social reality a purely situational product but recognizes societal building blocks and thus societal order as the ingredients out of which practices are produced that create and maintain social reality. The strong data focus of some micro-analytical studies is prone to disregard the importance of broader contexts that exceed the here and now for an emic understanding of the phenomena under study, to which we will come back in the section “Methodological Implications.”

Having established that environments are not ontic facts but epistemic tools of an observer does not imply that they are foreign to the inside views of a group. On the contrary, since the environments are conceived of as correlates of the social processes that group members are involved in, the description of a particular group environment has to pay tribute to their emic perspectives. As self-descriptions of groups might not literally include an ecological model, the environments have to be conceptualized on an abstract level that allows for specifications in different cultural and historical contexts. The ecological model has the advantage of offering both descriptions of social reality as it appears to groups and comparability by organizing these descriptions around environmental parameters broad enough to be applied to different lifeworlds.

There is no finite number of environments of social processes. For the purpose of examining processes of group identity fabrication, the author conceptualized eight environments: psychic-personal, semiotic-medial, technical, institutional, emotional, spatial, discursive, and groupal environments. There is no space here to discuss all environments (see Kurilla, 2020a). Instead, institutional environments will be addressed briefly as an example^18^.

In social sciences, institutions are often described as either sources (Gehlen, 1977, 2004; Hobbes, 1998) or limitations (Adorno, 1967; Dahrendorf, 2006) of human freedom. Institutions indeed have a double aspect. They constitute the riverbed that both lays the foundations for and limits social practice. As a result, both Gehlen’s credo that institutions “relieve” us and Adorno’s critique that they work against human freedom can be affirmed. The concept of institutions is neither bound to society nor to other collectives and can thus be employed in the study of groups. The institutional configuration of a group can be considered its culture, which echoes Gehlen’s (1977) conception of culture as nature modified by action.

Institutions can be relevant as frames of group interaction. It makes a difference whether a group acts as a limited company, club, or matrimony. Institutions are not always legally binding. Relevant for group processes may be the etiquette, customs, rituals, communicative genres, or, generally, interaction models. Some interaction models become characteristic for groups and enable or limit the communication with other groups. Helmolt (1997) shows that French teams often operate with the help of the fraternizing form of “complicité,” which can lead to problems in the cooperation with German teams to whom this interaction form is unknown. Forms may be entirely unknown to other groups or hard to put into practice if a certain relevance pattern does not fit the participant such as in the case of “secretarial bitching” that Sotirin and Gottfried (1999) describe.

Environments and the raw materials obtained from them are emic fabrications of the group. The societal character of institutions such as love or marriage stems from the fact that groups entertain relationships to the exterior, as a result of which their fabrications cannot be entirely arbitrary. This becomes particularly evident in the case of intergroup conflicts. A group’s models of antagonism (see Kurilla, 2013) have to be adaptable in order to entertain conflictive relationships with other groups. It makes a difference whether we quarrel, fight, or just discuss a matter, which of course varies according to, e.g., regional parameters. Unlike in Luhmann’s view, love is not regarded a generalized medium of communication that, on a global scale, serves to construct intimate relationships through personal communication. Love is rather an institution of groups with parallels to similar institutions in other groups. Cultural differences of love are well documented (e.g., Averill, 1985; Mees and Rohde-Höft, 2000; Schröder, 2004; Scheff, 2011). The same is true for friendship, unlike Kühl’s (2021b) narrow concept of groups might suggest. The concept varies locally and historically. A friendship ethos may belong to the past in Central Europe (Luhmann, 1983), in other regions such as the Spanish Basque Country it still governs everyday relations, is conceptually nuanced, carries highly binding expectations comparable to formal organization, and leads to spatial institutions such as gastronomic “sociedades.”

During the constitutive stages of a group, its institutional components are not always present as objectifications. When an objectified identity is eventually formed, practices retrospectively turn into practical identities. Being lovers or “doing love” can be a pre-reflective practice that not even for the lovers is objectified as such. The constituting group may ask itself, “are we dating,” or “are we still dating,” which evidently can also be brought up by others. The communicative treatment of those questions highlights the options of objectifications the partners share. Not all groups form environments where “dating,” “polyamory,” “polygamous” or “monogamous marriages” are options for group constitution. Like all group environments, pre-reflective and objectified institutional environments and their histories of being transformed into process components are constituted during group constitution and cannot be reduced to residues of individual socializations. They are genuine fabrications of emergent social processes in groups.

## Methodological Implications

The communication-ecological approach establishes an abstract foundation-theoretical position that is to be concretized during empirical studies. It helps to depict everyday life phenomena faithfully, as they are not forced into a narrow conceptual corset. Simultaneously, heterogeneous phenomena from different lifeworlds are rendered comparable, as they are captured with a homogeneous theoretical base. The methodological departure point lies in the ethnographic comprehension of everyday realities without prematurely classifying their elements with categories derived *ex ante*.

The analytical differentiation of group realities into environments of social processes can serve as the grounds for thick descriptions (Geertz, 1987). The model of analytically differentiated environments helps to organize and thus to render comparable everyday practices and provinces of meaning. The communication-ecological model is able to depict everyday life in a way that is faithful to the inside views of groups and individuals and at the same time makes it accessible to scholarly discourses, which are the two main ingredients of thick descriptions.

As in Kelle’s (1997) notion of an “empirisch begründete Theoriebildung” that builds on Strauss and Corbin (1998) and Glaser and Strauss (2009), categories and hypotheses are not developed *ex ante* but also not simply extracted from the data material. They rather result during research from an abductive (Peirce, 1978, 1979) operation that mediates between empirical data and an open theoretical frame and thus contributes to the “quasi-inductive evolution of science” in the sense of Popper (2002). As has been shown above (section “Environments of Group Communication”), the notion of institutional environments sensitizes for institutional components of groups without forcing the phenomena into too narrow or, from an emic viewpoint, inappropriate categories. Additional insights can be generated by examining other environments such as semiotic-medial, discursive, spatial, emotional, psychic-personal, technical, and groupal environments. Focusing on individual environments does not inadequately obscure the role of other environments, as environments are only analytically distinguishable and their interrelatedness in empirical phenomena can be traced in orientation on the communication-ecological model as a whole. The option of examining the environments individually provides researchers with the opportunity to focus on their particular research interest without losing sight for the bigger picture and/or *a priori* neglecting social and cultural differences.

In view of the double aspect of environments as correlates of pre-reflective practices and as objectifications, there are some methodological particularities. Beside participant observation, narrations provide a way of accessing foreign lifeworlds. No matter what their semiotic-medial manifestations and interactive fabrications might be, however, narrations objectify the practice they portray to a certain degree. Narrations can nevertheless entail pre-reflective relations. To synthesize these relations, not the figure of the narrations but their backgrounds have to be examined (Kurilla, 2013, 2020b). Under this condition, narrative interviews deliver a research-pragmatic substitute for participant observation. The *readiness-to-hand* (Heidegger, 1967) becomes tangible in narrations as silent relations within practical contexts.

Practical meaning is constituted in relation to action contexts and is thus not present in an objectified way. These contexts have to be taken into account when fabricating audio-visual recordings, transcriptions, and, even more so, during data analysis. Micro-analytical studies in the tradition of the ethnomethodological conversation analysis trade this focus on action contexts for a strong focus on empirical data obtained through technological recording devices. Like in positivism, extensive data collections are built up in a presumably unbiased way, without supposedly misleading preconceptions of everyday life to extract general regularities that are seemingly immanent in the data material (Flader and Trotha, 1988; Ehlich, 2007). Interpretation processes that guide the transcription of recorded documents often remain disregarded and are thus not systematically taken into account to improve the process of analysis and its conceptual depiction. Closer examination even reveals that conversation analysis does not only suffer from a “secret positivism” (Flader and Trotha, 1988) but sometimes even comes close to a sensualist epistemology because, unlike in positivism, it is not assumed that the general can be inferred from individual cases. The underlying assumption rather seems to be that the general is identical with the factual (ibid.). The researchers’ own interpretation performances disappear in the blind spot of their observations, which leads to an epistemological self-misconception, as the theoretical premises necessarily employed in the process of knowledge generation are not taken into account, let alone reflexively explicated.

The communication-ecological model helps to prevent such epistemological derailments by methodically transcending the here and now of situational data. Orienting on analytically separated environments on an abstract level that have to be concretized during empirical studies, researchers become sensitized for their own preconceptions that inevitably influence the production and analysis of data and, at the same time, for cultural and social differences among the subjects they study. With these premises, micro-analytical studies offer a promising way to shed light on process histories, i.e., the pre-reflective aspects of social processes in and among groups.

As pre-reflective social processes constitutive for groups are only recursively observable as such,^19^ it seems that research on these processes has to begin with retrospective narrative objectifications. The constitution of pre-reflective sense, however, can still be observed *in actu*. This requires a specific stratagem that Bühler (1978) employs in distinction from purely behaviorist approaches. The observation of practices operates in a hypothetical *as-if*-mode. This way, premature commitments to an interpretation are avoided. The orientation on an *as if* also helps to prevent neo-positivist self-misconceptions, since assumptions are inevitably explicated. Even the categories developed by Bales (1951) could be employed in the *as-if*-mode as long as they are considered hypothetical constructs that can be modified or completely abolished during research in view of individual group realities.

Be it zero-history or focus groups, all groups can serve as a research paradigm in the frame of the presented theory. Decisive is, however, that they are ethnographically observed. Should, e.g., focus groups be employed, attention has to be paid to the constitution of their environments to examine the emerging emic views. Counterintuitively, not despite their relative artificiality but because of it, focus groups even literally invite research on the constitution of environments of social processes in groups.

## Discussion

The conceptual devices to describe and explain group communication have been introduced in a conceptually coherent theoretical framework. This framework is transparent regarding its epistemological and conceptual premises and thus open to scrutiny. Communication has been described as an emergent process that is propelled by the ascription of messaging intentions. It has been differentiated from other social processes that are not communicative but influence communication. Different paradigms of communication in and among groups have been presented. The communication-ecological model has been implemented in an overarching theoretical framework that allows phenomena of group communication addressed by different approaches to be unified with the help of a coherent conceptual base. The theory is situated on such an abstract level that it can be concretised in view of empirical data across social and cultural differences.

The communication-ecological model is not only utterly coherent but also able to describe and explain the phenomena of group communication that the approaches discussed above focus on. The deliberating groups of functional theory that aim at rational decision making do exist in everyday life and can be described with the analytical tools presented here. These rather societal groups, however, do not limit of the understanding of groups, neither does the reduction of groups to communal groups such as friendships in the tradition of Luhmann. Unlike functional theory and Luhmann’s systems theory, the presented model does not *a priori* limit the range of phenomena and thus avoids ethnocentric biases.

It has been shown that symbolic convergence theory confounds two logical levels that in the presented theoretical frame are treated separately. Phenomena of order are not only explained as coherent chains of symbols that over time become more predictable but concern all process components. The reduction of contingency of future processes has been explained with the concept of process history and its recursive narrative objectification, avoiding the term “structure” to emphasize the distinction from Giddens’ structuration theory among others.

The attention the bona fide perspective pays to context relations has been systemized through an abstract model of different environments. Group boundaries have been considered epiphenomena of fabrication processes of objectified and practical identities. The communication-ecological model conserves the openness of this perspective without permitting an *anything goes* regarding the concept of communication and other conceptual devices.

It has also been shown that the presented model is able to unify research on intergroup communication with research on small groups in one framework. Sufficient distinctions between groups, their actual components, and possible fictional extensions have been drawn to clearly describe the phenomena under consideration. Alternative concepts of interpersonal and personal communication have been offered that are more adaptable to the common distinctions in communication research.

Efforts have been undertaken to establish theoretical coherence and terminological precision regarding identity concepts. Group identities have been depicted as carried by social processes and not as psychical residues. A clear distinction of different identity types has been offered. Individual and group identities have been divided into objectified and practical aspects, the latter of which can only be identified retrospectively. Collective identities were considered the materials individual and group identities are fabricated of and thus placed on another level of analysis.

The communication-ecological model allows for faithful depictions of the emic views of groups and, simultaneously, for comparisons of different lifeworlds. Environments are situated on an abstract level that allows for concretizations across social and cultural differences. The emphasis that the empirical description of environments has to match the inside views of groups prevents researchers from a positivist self-misconception. The model is based on a broad understanding of groups, which contributes to its openness for social and cultural differences. Unlike in Luhmann’s view, environments are not bifurcative correlates or an empty surplus of processes that enable future operations. They bear sense in a double meaning – objectified and pre-reflective sense.

The whole theory avoids the Cartesian reductionism by consequently taking into account the difference between objectified and pre-reflective processes. This does not only render its descriptions of everyday life where reflective thought is everything but the norm more faithful. It also helps to integrate the theoretical insights of authors like Merleau-Ponty (1995), Bourdieu (1996), Brandom (1998), Wittgenstein (2003), Polanyi (2009), etc., regarding phenomena of embodied practices, habitus, tacit knowledge, etc., that have not yet obtained a systematic place in social theory. The inclusion of pre-reflective processes undoubtedly increases the complexity of the theory. This increase, however, is required in order to be faithful to everyday life.

The difference between practice and objectification also governs the distinction of two different ways in which the contingency of social processes is reduced. On the one hand, inertia of social processes is established practically. This is called the *process history*. On the other hand, objectifications of past processes are fabricated that outline the space of propositional potentiality. This is termed the *narrative objectification* of process histories. Like in Walter Benjamin’s (1974) understanding of history, past and future are products of the here and now, as a result of which contingency reduction is the work of current processes. Process history and narrative objectifications occupy the space that, in social theory, is usually filled with concepts of structure. The distinction emphasizes that contingency is reduced on two levels, which is not even captured by Giddens’ “duality of structure.”

It is the author’s hope that the ecological model may contribute novel impulses to the paradigm discussion in the field of social theory. To take on this endeavor, the presented environments can be taken as a starting point. Studies may focus on how space, discourses, emotions, etc., are fabricated by social processes that take place in and among groups. Further environments may have to be added. The theory has to be concretized in view of research questions and empirical fields and can be expanded this way. In research on group communication, the advantage of combining different approaches in one overarching theoretical framework while avoiding conceptual difficulties surely outweighs the increase of complexity and facilitates novel cooperation and understanding among adherents of seemingly unconnected or incommensurable approaches.

## Data Availability Statement

The original contributions presented in the study are included in the article/supplementary material, further inquiries can be directed to the corresponding author.

## Author Contributions

RK drafted the manuscript and participated in the review and revision of the manuscript, and has approved the final manuscript to be published.

## Conflict of Interest

The author declares that the research was conducted in the absence of any commercial or financial relationships that could be construed as a potential conflict of interest.

## Publisher’s Note

All claims expressed in this article are solely those of the authors and do not necessarily represent those of their affiliated organizations, or those of the publisher, the editors and the reviewers. Any product that may be evaluated in this article, or claim that may be made by its manufacturer, is not guaranteed or endorsed by the publisher.

## Footnotes

1 Groups of two members form a special case, as the maximum is already realized with the minimum degree of interconnectedness.

2 In Plessner’s (1975) philosophical anthropology, humans are distinguished from other animals by their capability of not only moving in a given medium but also seeing themselves from the outside as being situated in their medium. In Plessner’s own words, humans are “excentrically positioned.” From this point of view, the group as understood here can be considered the example *par excellence* of human social life.

3 Marx (1977) himself uses the expressions a “Klasse gegenüber einer anderen Klasse” and a “Klasse gegenüber dem Kapital.”

4 See Kurilla (2020a) for comparisons to and differentiations from other authors such as James (1890), Sartre (1947, 1983), Marx (1977), Luhmann (1985), Lacan (1991, 2006a, b), and Hegel (2005).

5 Unlike in the Husserlian phenomenology, however, social life has a deciding influence on the constitution of phenomena in Meads thought.

6 Like the ego in Sartre’s (1997) view, the I is only accessible as a delayed construct, as it can only enter reflective thought retrospectively as an objectification.

7 Should a constituting group of face-to-face interacting individuals that identifies as *euskaldun*, however, imagine itself as being part of the bigger entity of “the Basque,” this equals an identification of the group with an “imagined community” (Anderson, 2006). This is a “fictional extension” of groups as long as the minimal conditions are not met to consider others who are not present as group members.

8 The fact that also group identities can be used to form individual identities is disregarded here for the purpose of simplifying illustration.

9 The following quote illustrates that collective identities do not only unite but also divide: “Scruncher. When I take over the world, I plan to exterminate all folders” (The Escapist Portal, 2012).

10 The value of this model for the engineering problem of transferring a physical signal through a channel with a given capacity and a noise source from a sender to a receiver are undeniable. This is, however, only a small part of communication processes. The reductionist depiction of communication as the transference of signals has been criticized by countless authors such as Juchem (1985), Ungeheuer (1987, 2004), Schmitz (1994, 1998, 2018), Luhmann (1998, 1999), Loenhoff (2002, 2010a, 2010b), Seel (2010).

11 Bühler’s “Steuerungsmodell” consists of two subsystems (A and B) with one sending device (S) and one reception device (E) each. Between the subsystems is a medium such as air. Various feedback loops are possible within the system as a whole and within the subsystems (such as proprioceptive feedback that is indicated with the dashed arrow from the periphery to the receiver E). The arrows between E and S do not indicate a mechanical relation between the reception and the sending device. Bühler rather describes contact phenomena as guidance over a synaptic cleft to emphasize the relative autonomy of the subsystems A and B. Considering the level of abstraction Bühler’s cybernetic model is situated on, it can be applied to organizations, institutions, states, etc., although it was originally developed to describe and explain interpersonal interaction.

12 Communication does not have to be based on verbal language, but verbal language is, according to Luhmann (1999), such an improbable arrangement that it is unlikely that no messaging intention is attributed when language is observed by others.

13 The speech act theory (Austin, 1962; Searle, 1969), e.g., suffers from the shortsightedness of reducing communication to processes governed by speaker intentions.

14 The “phenomenal intentionality research program” doubts that collectives are have a “phenomenal consciousness and underived intentionality” (Baddorf, 2017) characteristic for minds.

15 These categories may not be purely cognitive devices but tied to contexts and act as instruments of conversation (Edwards, 1991). Similarly, empirical studies show that identity categories are not purely objective societal products but emerge in conversation (Antaki and Widdicombe, 1998). This does, however, not affect the conception that the mutual ascription of different identities among two individuals does not suffice to describe their interaction as “intergroup communication.”

16 This example of for the third type intragroup communication does not undermine the micro-analytical research conducted on the interactive and material coordination of action (see, e.g., Engeström and Middleton, 1996; Hutchins and Palen, 1997; Suchman, 1997; Hindmarsh and Pilnick, 2002). These empirical studies concern, however, the second type of intragroup communication where members communicate with each other. The third type of intragroup communication is concerned with the case when the group as a whole communicates with the group as a whole. The shift from the second to the third type requires a change of perspective. This change has been made possible by a focus not on messaging intentions but on the ascription of messaging intentions to individuals as well as social entities. The group as a whole may not have messaging intentions but they can be ascribed to the group as a whole, which is why it can act as an address of communication.

17 This conception bears similarities to Wenger’s (1999) concept of a “dual constitution of histories.” Wegner’s distinction of participation and reification, however, is not to be confounded with the distinction of process histories and narrative objectifications, as the latter is directly derived from Heidegger’s (1967) distinction of *ready-to-hand* and *present-at-hand*.

18 Institutional environments have been chosen as an example, as they shed light on social and cultural differences in the constitution of groups in view of available emic models, which concerns the main interest of this article. A look at technical, emotional, spatial, and particularly semiotic-medial environments would have placed more emphasis on the material, multimodal, and embodied aspects of social processes in and among groups (see, e.g., Orr, 1996; Loenhoff, 2001; Goodwin, 2007; Streeck et al., 2011). A thorough consideration of these aspects, however, would have exceeded the scope of this article.

19 See the discussion of practical group identities in the section “Identities.”
